# Supplementary material for: Differences in Charge Distribution in Leishmania tarentolae Leishmanolysin Result in a Reduced Enzymatic Activity
Source: Int J Mol Sci. 2022 Jul 11;23(14):7660. doi: 10.3390/ijms23147660 (PMC9321319; doi:10.3390/ijms23147660)
Supplement: Supplementary file 1 [file ijms-23-07660-s001.zip › Supplementary Tables and Figure Legends.pdf]

Supplementary Material – Tables

Table S1. *In silico* analysis of leishmanolysin sequences from *Leishmania tarentolae* and the crystalized LmjF.10.0460 leishmanolysin from *L. major* retrieved from TriTrypDB.

Excel file

Table S2. Evaluation of *L. major* (1LML) and *L. tarentolae* (LtaP10.0480, LtaP10.0650, and LtaPcontig00616) leishmanolysin proteins.

**Models**	**ERRAT**	**QMean**	**MolProbity**	**Ramachandran Plot**			
				**R1**	**R2**	**R3**	**R4**
**LmjF.10.0460 (1LML)**	92.1	0.03	1.48	89.8	9.8	0.2	0.2
**LtaP10.0650**	87.6	-1.12	2.13	91.0	8.3	0.2	0.5
**LtaP10.0650_ref**	92.09	0.77	0.87	91.7	7.8	0.5	0.0
**LtaP10.0480**	86.6	-1.66	2.51	90.5	8.6	0.7	0.2
**LtaP10.0480_ref**	88.36	0.57	0.71	93.6	5.9	0.5	0.0
**LtaPcontig00616-1**	81.40	-2.27	2.53	89.8	8.9	0.8	0.5
**LtaPcontig00616-1_ref**	84.41	-1.82	0.85	92.1	7.6	0.3	0.0

R1: residues in most favored regions; R2: residues in additional allowed regions; R3: residues in generously allowed regions; R4: residues in disallowed regions.

Table S3. Aminoacid composition and protein total charge of leishmanolysin 3D structures of *L. major* (1LML), *L. tarentolae* (LtaP10.0480, LtaP10.0650, and LtaPcontig00616), *L. braziliensis* (LbrM.10.0600), and *L. martiniquensis* (LMARLEM2494_100009500) at pH 5.5 and pH 7.4.

**Protein**	**Negative (Asp + Glu)**	**Positive (Arg + Lys)**	**Protein total charge**	
			**pH 5.5**	**pH 7.4**
1LML	51 (10.7 %)	34 (07.1%)	-6	-16
LtaP10.0480	46 (09.4%)	50 (10,3%)	13	4
LtaP10.0650	51 (10.5%)	43 (08,8%)	-1	-7
LtaPcontig00616-1	49 (11.0%)	45 (10.1%)	7	-4
LbrM.10.0600	54 (11.3%)	37 (07.7%)	-11	---
LMARLEM2494_100009500	52 (11.0%)	40 (08.5%)	-6	---

Supplementary Material – Figure legends

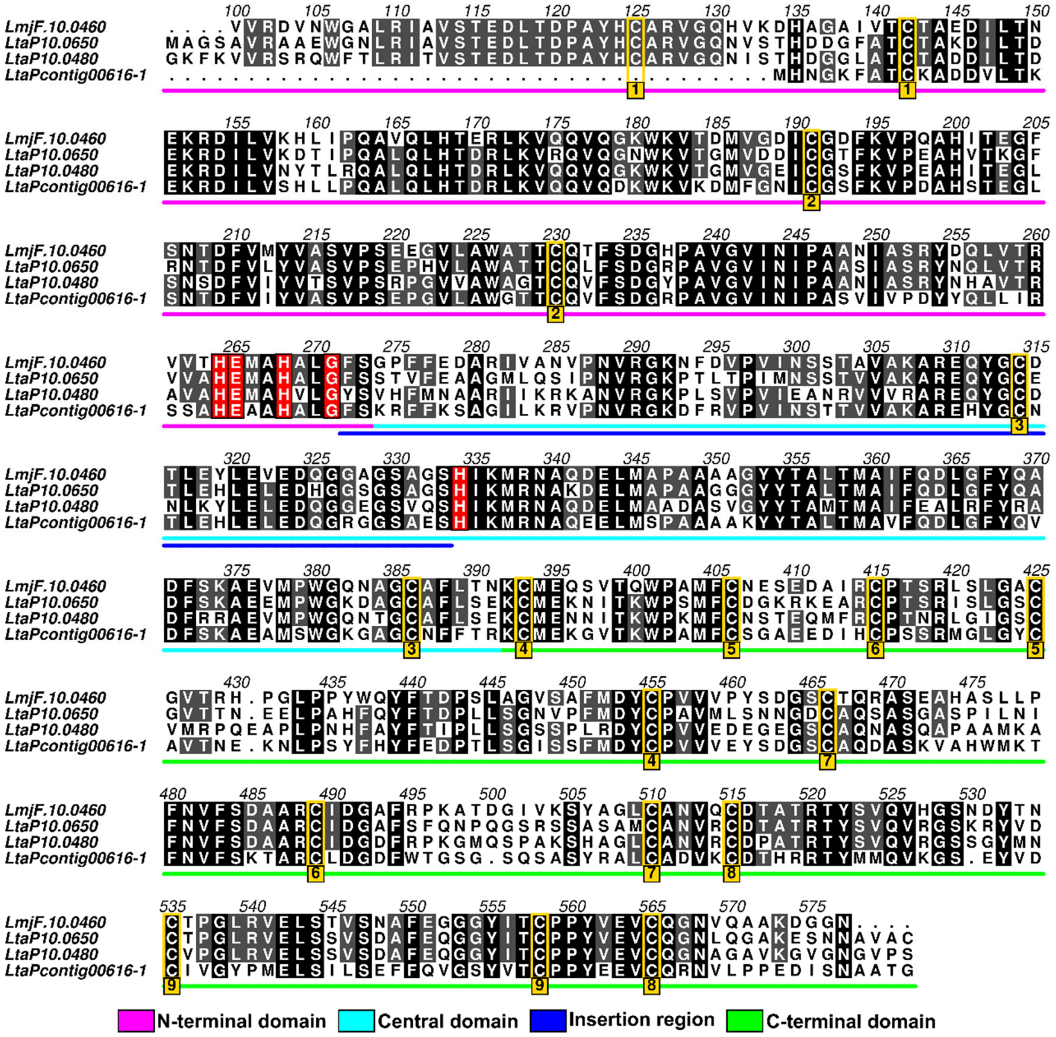

Figure S1. Multiple sequence alignment of *L. tarentolae* leishmanolysins employed for the comparative modeling based on the structure of *L. major* leishmanolysin (PDB ID: 1LML). Black and gray filled positions in the sequence alignment represent fully and partially conserved residues, respectively. Gaps are represented by points. The N-terminal, central region, and C-terminal domains are underlined in pink, light blue and green, respectively. The Motif HExxHxxGxxH is highlighted in red and the insertion region is underlined in dark blue. Cysteine residues that form disulfide bonds are highlighted in yellow.

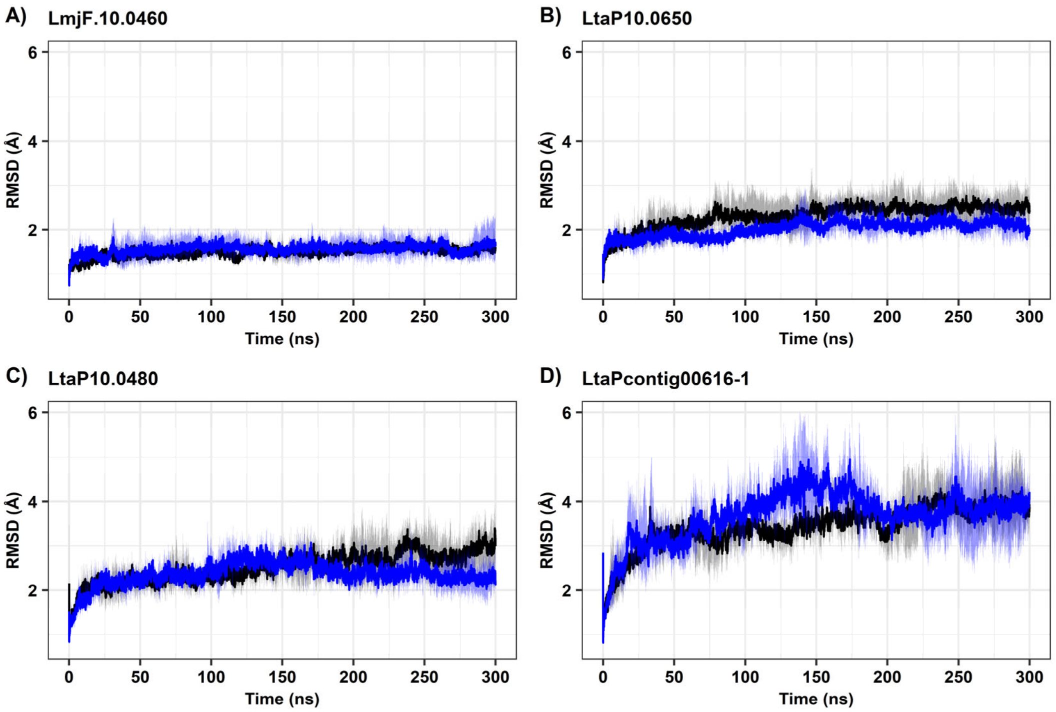

Figure S2. Root-mean-square deviations (RMSD) of *L. major* and *L. tarentolae* leishmanolysins at different pH values of 5.5 (black) and 7.4 (blue). The Mean and the confidence interval of the replicates are displayed as solid lines and smooth lines, respectively.

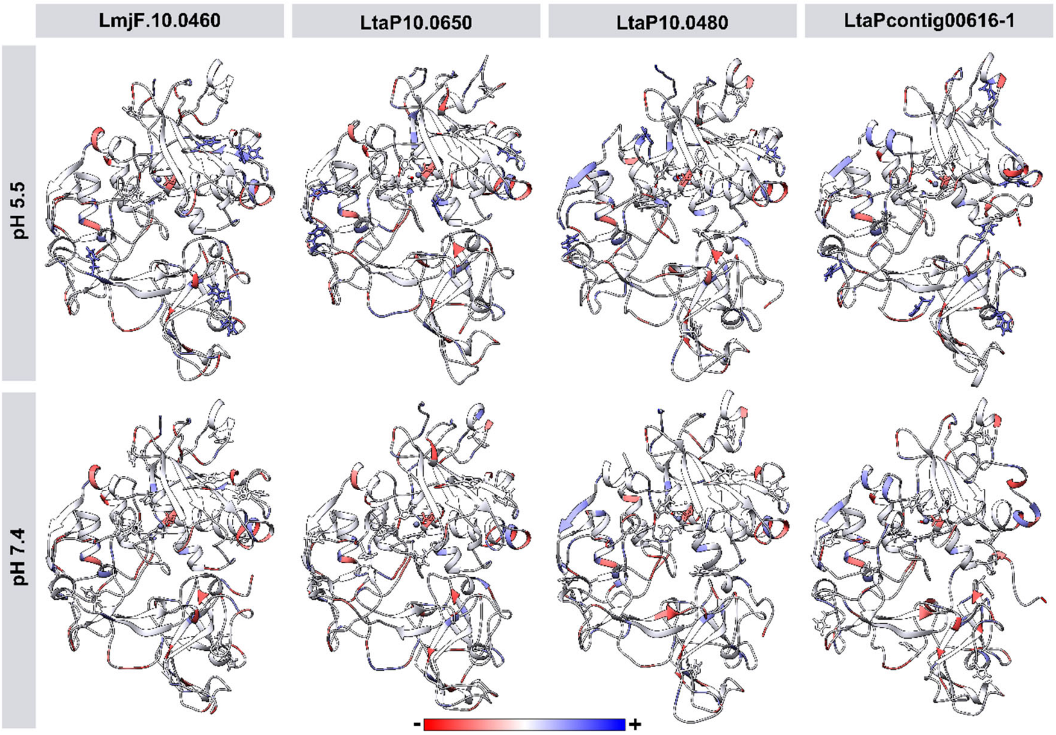

Figure S3. Three-dimensional structures of *L. major* and *L. tarentolae* leishmanolysins colored according to the atomic charge for pH values of 5.5 and 7.4. Sticks represent the zinc-binding motif HExxHxxGxxH and the histidine residues that had protonation change according to the pH values.

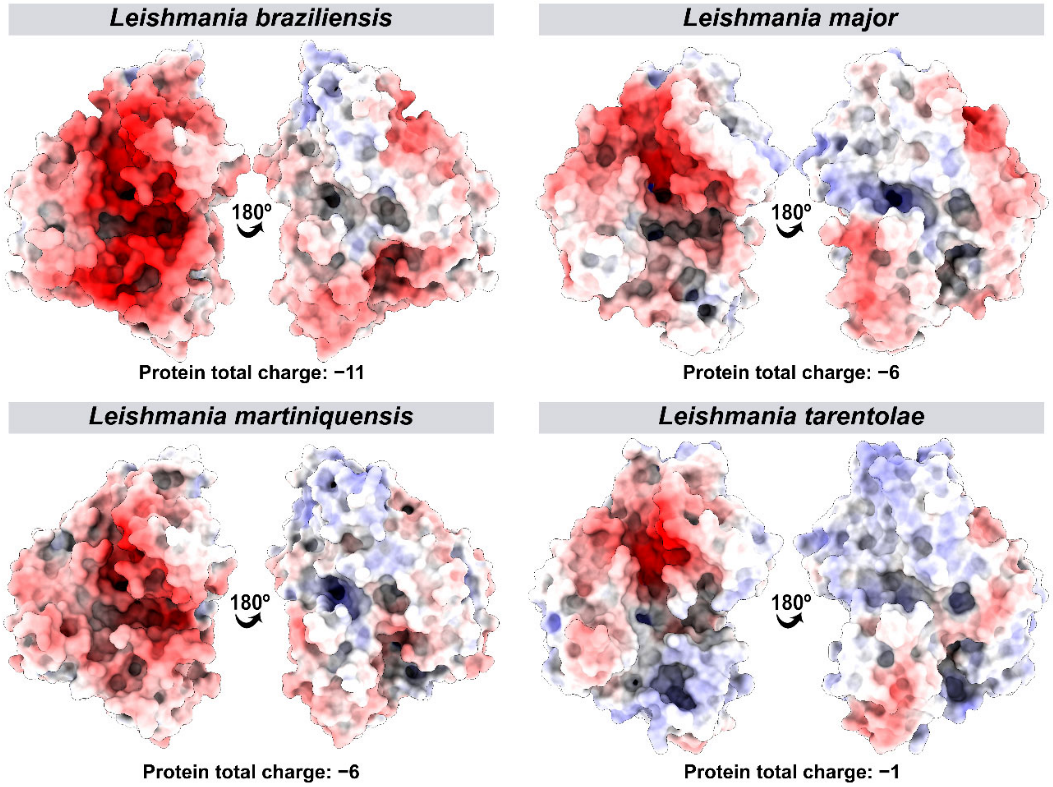

Figure S4. Differences in the surface electrostatic potential of leishmanolysins from *L. braziliensis*, *L. major*, *L. martiniquensis*, and *L. tarentolae*. The comparative molecular dynamic analysis was performed using the crystal structure of *L. major* LmjF.10.0460 (PDBid: 1LML) and the modelled *L. braziliensis* LbrM.10.0600, *L. martiniquensis* LMARLEM2494_100009500, and *L. tarentolae* LtaP10.0480, LtaP10.0650, and LtaPcontig00616_1, in pH 5.5. The molecular surface is colored according to electrostatic potential using the Chimera software; where red, white and blue correspond to acidic, neutral and basic potentials, respectively.

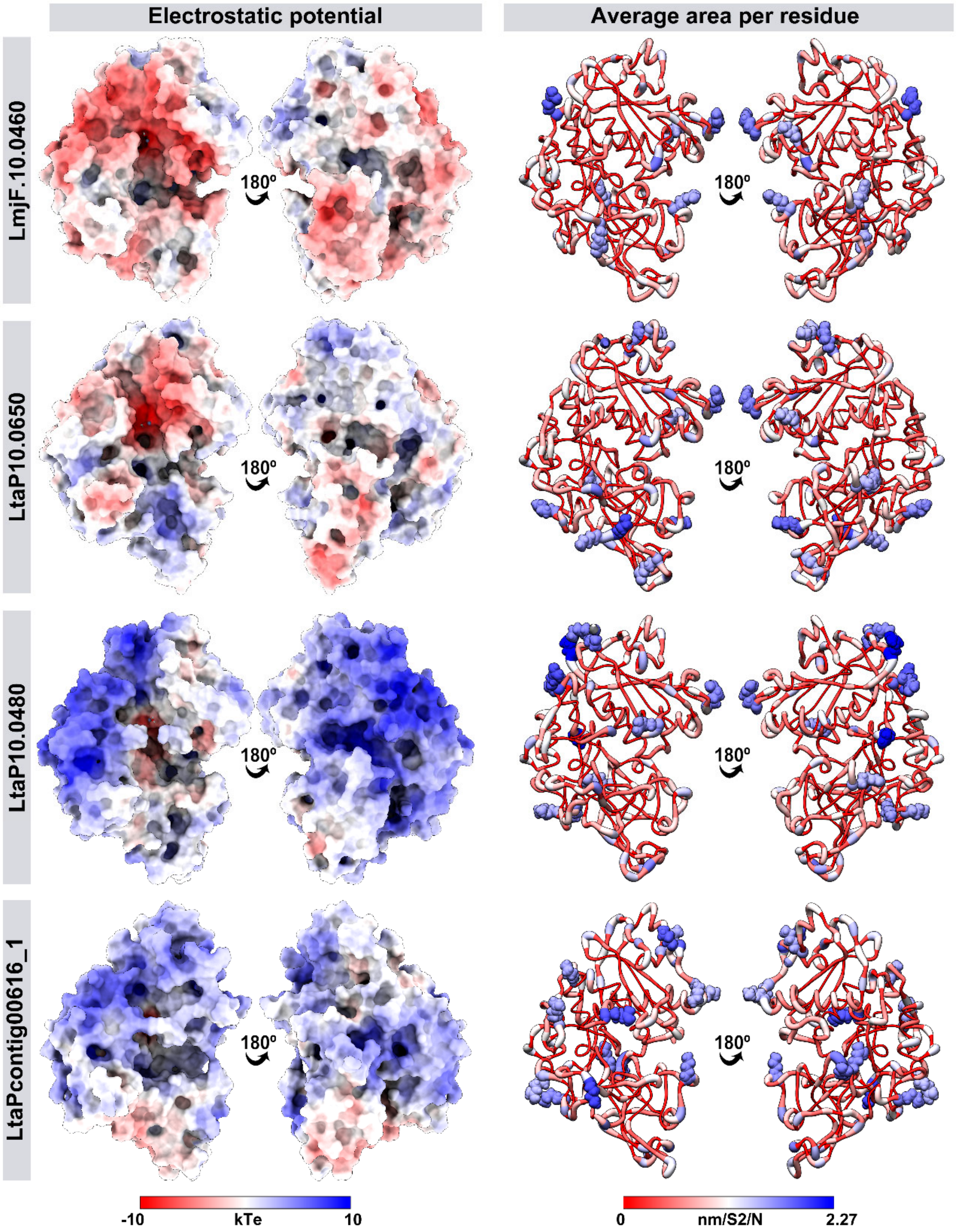

Figure S5. Comparison between electrostatic potential (pH 5.5) and residue solvent accessibility of *L. major* and *L. tarentolae* leishmanolysin 3D-structures. The molecular surface and residue solvent accessibility are colored according to the figure caption. Lysine and arginine with solvent accessibility >1.5 nm/S2/N are represented in CPK (sphere of radius equal to the van der Waals radius).

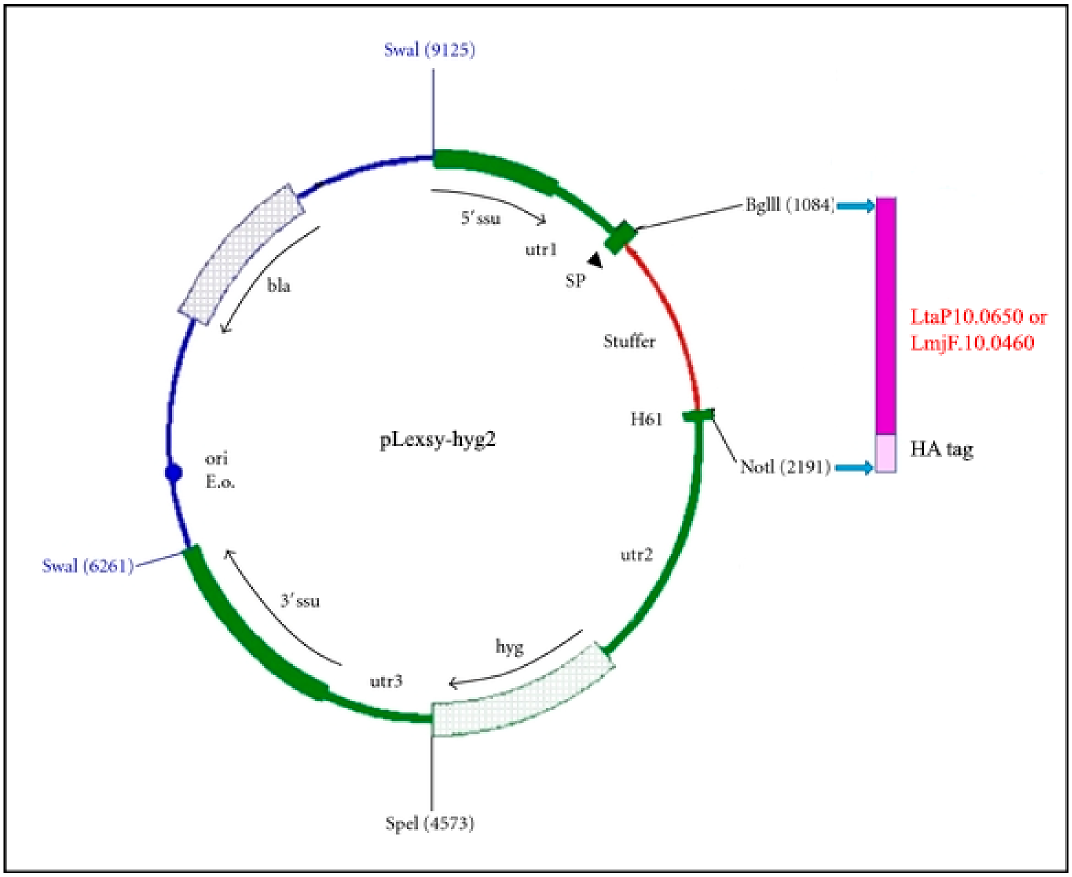

Figure S6. Graphical overview of pLEXSY-hyg2 (EGE-232) plasmid construction containing *L. tarentolae* and *L. major* leishmanolysin sequences. Expression vector used to clone *L. major* LmjF.10.0460 and *L. tarentolae* LtaP10.0650 genes are shown. The cDNA encoding leishmanolysin sequences was inserted in corresponding restriction sites by SwaI, BglII and NotI. The plasmids were transfected by electroporation in *L. tarentolae* Parrot-TarII strain ATCC PRA-229.

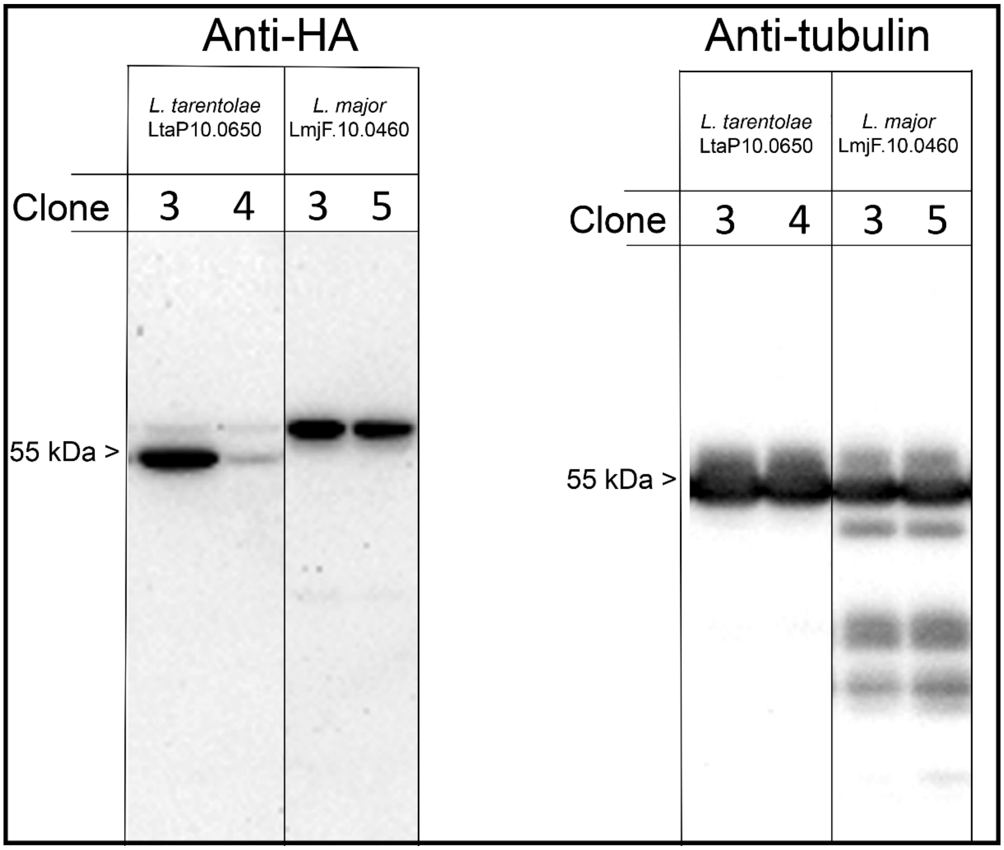

Figure S7. Western blotting analysis of transfected *L. tarentolae* clones after selection with hygromycin B. The soluble extracts of *L. tarentolae* transfected clones with *L. major* LmjF.10.0460 and *L. tarentolae* LtaP10.0650 genes were submitted SDS-PAGE electrophoresis and transferred to a nitrocellulose membrane. The membranes were incubated with anti-HA antibodies and revealed by chemiluminescence. An α-tubulin antibody was used as load control. The numbers on the left indicate apparent molecular masses of the active bands expressed in kDa.

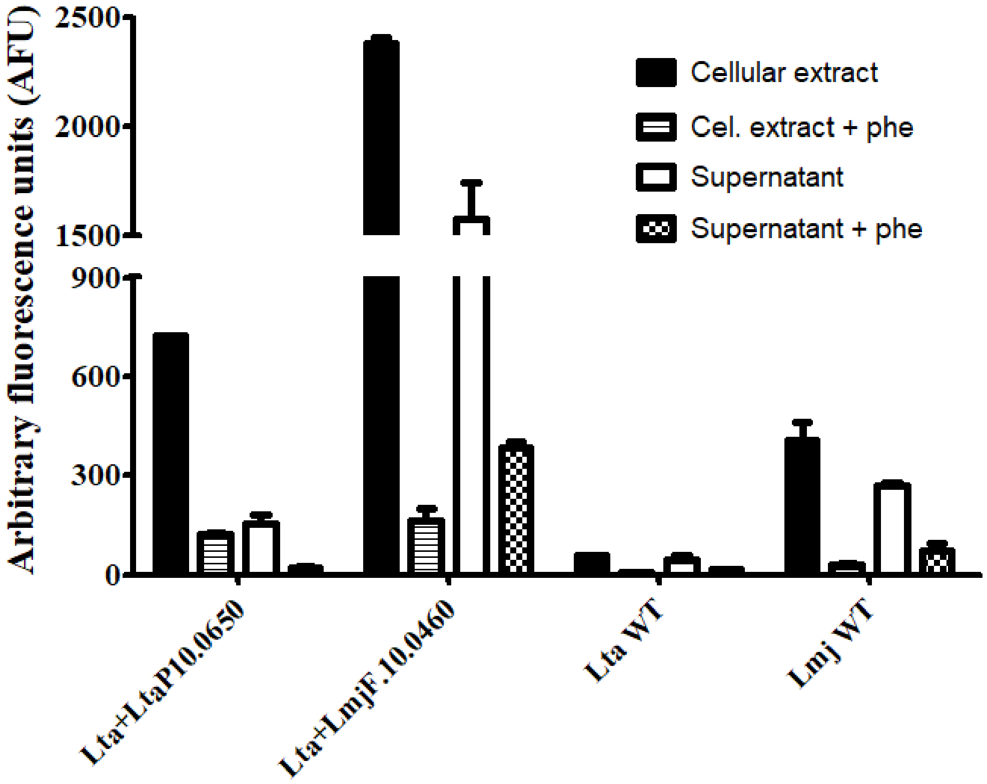

Figure S8. In solution proteolytic activity of the supernatant extract of overexpressed *L. major* LmjF.10.0460 and *L. tarentolae* LtaP10.0650 strains. The enzymatic activity was assessed by measuring the hydrolysis of the fluorogenic substrate MCA-Pro-Cha-Gly-Nva-His-Ala-Dpa-NH2 at 37 °C for 1 hour. Activity of the supernatant from wild type *L. tarentolae* (*Lta* WT) and *L. major* (*Lmj* WT), as well as the cloned leishmanolysin from *L. tarentolae* (*Lta*+LtaP10.0650) and from *L. major* (*Lta*+LmjF.10.0460) were measured in 100 mM glycine-NaOH buffer pH 10.0 buffer the presence or absence of 1 µM 1,10-phenantroline (*Lta*+LtaP10.0650 + phe and *Lta*+LmjF.10.0460 + phe) . Results are expressed as relative fluorescence units (RFU) and the values represent the mean ± standard deviation of three independent experiments performed in triplicate.
